# Migration infrastructures and the production of migrants’ irregularity in Japan and the United Kingdom

**DOI:** 10.1186/s40878-021-00242-4

**Published:** 2021-08-02

**Authors:** Nando Sigona, Jotaro Kato, Irina Kuznetsova

**Affiliations:** 1 Department of Social Policy, Sociology and Criminology, University of Birmingham, Edgbaston, Birmingham, B15 2TT UK; 2 Institute of Asia-Pacific Studies, Waseda University, Nishi-Waseda Bldg.7F, 1-21-1 Nishi-Waseda, Shinjyuku-ku, Tokyo, Japan; 3 School of Geography, Earth and Environmental Sciences, University of Birmingham, Edgbaston, Birmingham, B15 2TT UK

**Keywords:** Migration infrastructure, Japan, United Kingdom, Irregular migration, Immigration enforcement, Informal labour, Migration policy

## Abstract

The article examines the migration infrastructures and pathways through which migrants move into, through and out of irregular status in Japan and the UK and how these infrastructures uniquely shape their migrant experiences of irregularity at key stages of their migration projects.

Our analysis brings together two bodies of migration scholarship, namely critical work on the social and legal production of illegality and the impact of legal violence on the lives of immigrants with precarious legal status, and on the role of migration infrastructures in shaping mobility pathways.

Drawing upon in-depth qualitative interviews with irregular and precarious migrants in Japan and the UK collected over a ten-year period, this article develops a three-pronged analysis of the infrastructures of irregularity, focusing on infrastructures of entry, settlement and exit, casting a comparative light on the mechanisms that produce precarious and expendable migrant lives in relation to access to labour and labour conditions, access and quality of housing and law enforcement, and how migrants adapt, cope, resist or eventually are overpowered by them.

## Introduction

Japan and the United Kingdom (UK) face similar economic challenges, which are exacerbated by labour shortages and ageing societies (Ozgen et al., 2019). Historically, patterns of migration to and from Japan and the UK up to the end of World War 2 were largely shaped by the economic, political, and legal effects of colonialism, imperial expansion and retraction, and geopolitical adjustments and frictions linked to them (Hirota et al., 2019). Often, they occurred within the territorial boundaries of empire and would in current parlance fall under the internal migration rubric. In the post WW2 era, the UK experienced large-scale immigration from former colonies and Europe, while Japan kept its borders relatively closed. However, currently both countries are accepting similar numbers of migrants per year. According to OECD (2020) statistics, the UK has the fifth highest number of incoming migrants, 486,452 in 2018, with Japan ranked third, with 519,683. For both countries, at least until the 2020 COVID-19 pandemic, there is an upwards trend in immigration flows, however Japan has a lower stock of immigrants - approximately 2.8 million against an estimated 7.8 million in the UK. A fraction, yet highly visible in media and political discourse, of this immigration in both countries is unauthorised, at the point of entering the country or becoming so once in the country. For Gonzales et al. (2019, p. 3), the heightened visibility of irregular migration instigates many Western nations to put in place ‘immigration controls at an unprecedented scale to deter migration and to punish immigrants’, these measures affects not only new comers’ migration projects and entry routes, but shapes the everyday experiences of those already living in the countries.

The International Organisation of Migration (IOM) defines irregular migration as a ‘movement of persons that takes place outside the laws, regulations, or international agreements governing the entry into or exit from the state of origin, transit or destination’ (IOM, 2019, p. 116). This definition emphasises the conditions and circumstances of mobility and in particular of entry in a country. However, this is not sufficient to understand the phenomenon of irregular migration, as it leaves aside the reality of living in a country without papers, where the emphasis falls instead more on the right to stay and work in a particular country at a particular time. Düvell (2011) and Ruhs and Anderson (2006) reject the conventional, static, dichotomous migration status of being either legal or illegal. Düvell (2011) argues for a concept of ‘paths into irregularity’, while Ruhs and Anderson (2006) speak to the notion of different levels of ‘compliance’, which underline the spectrum of statuses and rights of individuals without papers vis-á-vis the state (Bloch et al., 2014). Overall, the line between authorized and unauthorized immigration is fluid (Yamamoto, 2007) and we argue that greater attention should be placed on the factors that produce specific configurations of ‘irregularity’, including the law (De Genova, 2002; Dauvergne, 2009; Lewis et al., 2014).

To inform the analysis of irregular migration and the factors shaping immigrants’ mobility pathways and entry and settlement opportunities as well experiences of immigration enforcement, this article brings together two bodies of migration scholarship, respectively: critical work on the social and legal production of illegality (De Genova, 2002) and the impact of legal violence on the lives of irregularised immigrants (Menjívar & Abrego, 2012; Bloch et al., 2014; Sigona, 2012; Round & Kuznetsova, 2016); and the role of migration infrastructures (Lin et al., 2017; Xiang & Lindquist, 2014, 2018) in shaping migration projects and trajectories.

There is relatively little research available in Japan and the UK on the diverse pathways that see immigrants move into irregular status, especially on how changes in immigration rules and regulations impact differently upon certain groups of immigrants, and what spaces for manoeuvre they may have in such circumstances. Furthermore, there is even less research on how immigrants, in both countries, cope on an everyday level with the move into irregularity, be this the experience of work, accessing health care, issues with law enforcement or family relations (for the UK, see Sigona & Hughes, 2012; Düvell et al., 2018).

Drawing upon in-depth qualitative interviews with irregular and precarious migrants in Japan and the UK collected over a ten-year period, this article examines the infrastructures and mechanisms that produce and reproduce irregularity, and how they shape the lives of irregular migrants at different points in their migration projects. It casts light on how, in both countries, irregular migrants adapt, cope, resist or eventually are overpowered by them. To capture the processual nature of irregularity production the examination will be focused on three stages of production, focusing respectively on the encounter of irregular migrants with infrastructures of *entry*, *settlement* and *exit*.

## Migration infrastructures and irregular migration

The act of migration cannot be reduced to a set of individual or household choices. Human mobility is heavily regulated through multiple interactions and different and often distinct governing contexts. Nowadays, opportunities for authorised mobility have shrunk (Dauvergne, 2009). Increasingly some migrants and some forms of mobility find themselves irregularised. The category ‘illegal immigrant’ therefore is only meaningful in relation to the contexts and circumstances that define it’ (Gonzales et al., 2019: 16). In other words, what counts as irregular migration and who is considered an irregular migrant varies over time and space and is embedded in specific conditions, histories, and structures of power (Ngai, 2014). While irregularity is rooted in legal classifications, its power to limit social mobility and narrowly confine everyday life lies not just in law but also in the discourses, politics, and practices which accompany the interpretation and implementation of laws (De Genova, 2002).

This approach accords primacy to the state and its apparatus in producing ‘illegality’, due to its power to define and police the boundaries of membership. Less attention is paid to non-state actors and to the interplay between different national, sub- and supra- national actors and processes (Gonzales et al., 2019) and how irregular migrants navigate this complex and dynamic environment and the opportunities that it provides (Chauvin & Garcés-Mascareñas, 2014). By adopting an infrastructural approach to understand the production of irregularity and its effects on irregularised immigrants, we will be able to account for a broader range of actors, agendas and interactions within and beyond the state apparatus. This expanded understanding of what and how irregularity comes into being in Japan and the UK, offers also insights into discursive and political spaces where immigrant agency can be meaningfully located (Sassen, 2002).

International migration is shaped by a series of interrelated and intensively mediated processes (Castles, 2004). The significance of ‘dematerialising’ connections, the growing role of ICT technologies and convergence between transport and communication (Sheller & Urry, 2006) as well as the role both of individual states and of changing international regulatory and surveillance administrations have been emphasised by scholars promoting the new mobility paradigm in migration studies (Glick Schiller & Salazar, 2013). Migration infrastructures have become one the main analytical tools to investigate and interpret human mobility in the context of East and Southeast Asia (Lin et al., 2017; Xiang & Lindquist, 2018), partly because the role of migration infrastructures is particularly visible in the region due to the historically significant role of private recruitment brokers (Lindquist, 2017) and state-driven emigration (Lee, 2017). Infrastructures are ‘always already inscribed with planning power, which dictates who gets or does not get to benefit from their socio-material arrangements’ (Lin et al., 2017, p. 3). Naturalised as taken-for-granted systems, infrastructures perform politics in their daily use through specific configurations of actors, elements and their relations. For Lin et al., ‘by recognising migration as the contestational result of these moments in infrastructuring, it then becomes possible to appreciate what makes migrant mobilities ‘real’ and ‘noteworthy’ in the first place’ (Lin et al., 2017, p. 169). In researching the infrastructures of irregular migration in Japan and the UK, we examine three interlinked assemblages of discursive, material and bureaucratic infrastructures, institutions and actors that in interaction shape and condition not only mobility, but also settlement and exit.

Most work on migration infrastructures focus on processes of recruitment and mobility of migrant workers or students (Robertson, 2017). However, recent scholarship has turned the focus on settlement (Ambrosini, 2017), and ‘arrival infrastructures’ in urban settings in destination countries (Meeus et al., 2019, p. 2) emphasising ‘the continuous and manifold “infrastructuring practices” by a range of actors in urban settings, which create a multitude of “platforms of arrival and take-off” within, against, and beyond the infrastructures of the state’. Moreover, recent theoretical advances in the field have also highlighted both material and immaterial components of infrastructures, from laws, trade arrangements and public discourses to social networks, ICTs and transport infrastructures. We have built from these insights to develop our comparative analysis of irregular migration in Japan and the UK.

Our approach applies the logic of infrastructures to the examination of the processes of *entry* and *settlement* of irregular and precarious immigrants in Japan and the UK, and the factors that shape their everyday life in particular with reference to housing and access to labour market, working conditions and migrant livelihoods. In doing so, it brings to the fore the changeable and ongoing nature of irregularity and the mechanisms that continuously produce some migrants as precarious and expendable in Japan and the UK.

Moreover, through the examination of the infrastructures of immigration enforcement and *exit* we will offer insights into the politics of immigration control in Japan and the UK and its differential impact on the lives of irregular migrants, building on critical scholarship on deportability (De Genova, 2002), detainability (De Genova, 2007) and immigration control (Anderson, 2013). For De Genova (2002), deportability as the possibility of being deported, rather than deportation itself, is one of the defining characters of the condition of ‘illegality’ and shapes the everyday lives of irregular migrants in their country of migration. However, enforcement practices and everyday bordering vary considerably between states and within, with some groups of migrants, because of their ethnicity, gender, age or immigration pathway, more visible to the gaze of immigration authorities (Peutz & De Genova, 2010; Bloch et al., 2014; Sigona, 2012; Yuval-Davis et al., 2019). The ever-present fear of being detected and removed keeps migrants and their families in fear, and constrains their actions and involvement in community life. The ‘deportation machine’ (Goodman, 2020) sees an increasing involvement of private contractors, and a vast industry built around the movement of ‘illegal migrants’ has flourished in the last two decades (Andersson, 2014). Our research examines the impact of the privatisation and atomisation of immigration enforcement in Japan and the UK highlighting the role of regulatory and commercial infrastructures in shaping migrants’ experiences of immigration control.

## Irregular migration in Japan and the UK

### International labour migrants between ‘front’, ‘side’ and ‘back’ doors in Japan

Let me be clear: We are not pursuing what is commonly considered an immigration policy.

(Japanese Ex-Prime Minister Shinzo Abe, 2 November 2018)^1^

The Japanese government’s decision in 2018 to introduce the category of medium-skilled workers in 14 labour-shortage sectors, made a breaking news globally because of the Japan’s reputation as a country closed for foreign labour force. Still, the Japanese ex-prime minister did not want this step to be seen as an immigration policy. Despite the growing demand for foreign labourers since the late 1980s, before 2018 the entry was legally restricted only to high-skilled labour (Milly, 2020), which meant that the shortage of workers had to be met by ‘side doors’ and ‘back doors’ (Thränhardt, 1999). The ‘back doors’ included mostly immigrants who had overstayed temporary visas and made up about one fifth of the number of registered foreign residents in 1993 (Ministry of Justice, 2017). ‘Side doors’ include students, technical interns and Nikkei-jin - Japanese-Brazilian and Japanese-Peruvian.

The *Action Plan for Realizing Society against Crime* adopted in 2003 labelled those who overstayed visas as ‘potential criminals’ strengthening immigration enforcement measures succeeding in curbing the number of ‘illegal stayers’ from 219,418 in 2004 to 113,072 in 2009. For Park (2017, p. 78), Japan’s reluctance to accept foreign labour, particularly Koreans, has historical and racist roots. Japan ‘labelled ethnic minorities living in the country as aliens, denied them the freedom to enter their country of nationality, and categorised them as ‘deportable”.

Though they were ‘illegal stayers’, migrants without right to statistics could still register at the city office and hold ‘alien registration card’. However, from 2012 foreign residents are required to apply for a new electronic Residence Card (zairyū kādo) integrated into the local government residence database ‘allowing the state to keep a closer check on foreigners in Japan as they move house or change job’ (Morris-Suzuki, 2015, p. 80). Such reform resulted in ‘the drawing of a sharper and deeper line between ‘desirable’ foreigners in Japan (particularly immigrants with high levels of technical skill, etc.) whose lives will generally be made easier by the reforms, and those who are either deemed ‘undesirable’ or who are to be kept at the very outer circle of the system’ (Morris-Suzuki, 2015, p. 81). These actions deterred the arrival of new migrants and led to a further reduction in the population of ‘illegal stayers’. However, since 2015 the population has recorded a new growth mostly due to overstaying technical interns and refused asylum seekers, this occurred in what continues to be a hostile political and media environment for non-high skilled migrants, with migrants on technical internships and ‘fake’ refugees as a preferred target of negative media coverage (Kato et al., 2019).

### Making the UK hostile to ‘illegal migrants’

The aim is to create here in Britain a really hostile environment for illegal migration. (UK Prime Minister Theresa May, 25 May 2012)

The so-called ‘hostile environment’ policy was announced in 2012 by the UK government led by the Conservative party, but some aspects had already appeared in previous Labour governments, particularly in relation to the treatment of asylum seekers and the right to asylum (Flynn, 2005). Immigration enforcement by proxy is one of the features of the policy-driven ‘hostile environment’ which has gradually drawn in employers, doctors, landlords and banks in the task, penetrating into the fabric of the everyday lives of irregular migrants (Bloch et al., 2014), what Yuval-Davis et al. (2019) calls ‘everyday bordering’. The impact of everyday bordering on the lives of irregular migrants varies, according to age, gender, ethnicity and their entry routes. This is the case, for example, for children and young people without immigration status (Humphris & Sigona, 2019) and racialized minorities (Bloch et al., 2014). The ‘hostile environment’ policy was translated in the Immigration Acts of 2014 and 2016, which included numerous measures to prevent people from accessing employment, healthcare, housing, education, banking and other basic services. The 2014 Act requires private landlords to check the immigration status of tenants and temporary migrants. Banks must check against a database of known immigration offenders before opening a bank account. The Act created new powers to check the immigration status of driving licence applicants and revoke those of overstayers. Extending the hostile environment further, in the 2016 Immigration Act the UK government sought to refocus efforts on illegal working and give more power to enforce immigration laws, including new measures to make it easier to detect and remove unauthorised migrants (Consterdine, 2018; Yeo, 2020). However, while detention and removal represent more visible and overt practices of immigration enforcement, the novelty of the ‘hostile environment’ policy is the introduction and enhancement of a portfolio of less tangible, but arguably more pervasive, tools that assist in achieving broader enforcement goals. These ‘soft enforcement’ measures have widened enforcement efforts and dramatically increased their effectiveness (Gonzales et al., 2019). While retaining and even strengthening controls on entry through restricting access to visa and externalised border checks, the novelty of the strategy consists in greater attention being paid to internal controls which often means policing access to essential public services (Consterdine, 2018; Yuval-Davis et al., 2019).

## Methodology

Empirical data on the experiences of irregular migrants in Japan and the UK was collected via in-depth qualitative interviews. Participants in both case studies were recruited through a combination of purposive and snowball samplings, drawing upon different social and personal networks. All names in the paper are pseudonyms to protect the identity of informants.

The research in Japan was conducted between July 2017 and March 2020 and included semi-structured interviews with 38 irregular migrants. The selection criteria included the irregular status of labour workers, which could be expired technical internship, expired Japanese language training and marriage visas. Most of the respondents (22 out of 38) came from Southeast Asia including 11 people from Vietnam, seven from the Philippines, and four from Myanmar. Others migrated from India, Nepal, China, Sri Lanka, Bangladesh, Turkey, Peru, Bolivia, Mali, Uganda and Nigeria. Sixteen of 38 interviewees had also applied for asylum. There were 12 women and 26 men, aged between 20 and 59 years old. Twelve stakeholders such as employers and supervision organizations and supporters were interviewed to gain additional insights into the working of the infrastructures of irregularity.

Fieldwork took place in Tokyo and the Kanto area located approximately 100 km from Tokyo, because some of the irregular migrants felt safer far from Tokyo and less visible to immigration officers and police. A researcher visited the dormitory of interviewees, a farm where informants worked, and a local festival attended by participants.

The UK case study is informed by in-depth qualitative interviews with irregular migrants collected as part of two complementary collaborative research projects focusing, respectively, on irregular migrant children and families (Sigona & Hughes, 2012), and the social and economic lives of young irregular migrants in Britain (Bloch et al., 2009). The former involved the collection of 53 interviews with irregular migrant children and parents originally from Afghanistan, Brazil, Jamaica, China, and Kurds from Turkey, Iran and Iraq. For the latter, 75 interviews collected with irregular migrants from Zimbabwe, China, Ukraine, Brazil and Kurds from Turkey aged 18–31 years. For both studies, interviews were carried out in several languages by field researchers with knowledge of the languages spoken by participants. Interviews were successively transcribed and translated into English and analysed with the support of NVivo software. The interviews in the UK were carried out in Greater London, the Midlands and the North West. The UK case study draws on a subset of 48 interviews from these two studies. The sample, 36 women and 12 men, includes irregular migrant parents with children in the UK who entered the UK without authorisation (17) and visa holders who overstayed (31).

In the following pages, we present the empirical data for Japan and the UK in three sections examining, respectively, migrants’ pathways into irregularity looking at the infrastructures of entry, their experiences of settlement, focusing on labour and housing infrastructures, and finally, we analyse migrants’ experiences in facing immigration enforcement and control.

## Pathways into irregularity

How did irregular migrants arrive in Japan and the UK? How did they find themselves irregular? In addressing these questions, this section shows the diversity of experiences and trajectories concealed by the label ‘irregular migrant’ and the extent to which they are shaped by migration infrastructures.

### From technical interns and language students to irregular migrants

The main routes into irregular status in Japan are visa overstaying, failure to renew or change visa and, to a lesser extent, irregular entry. Among those who overstay their visa, the commercial and legal infrastructures for technical internship and language courses are common pathways into the country. To extend their legal stay in the country, some migrants with temporary visa apply for asylum, however this is only a temporary fix as Japan has one of the lowest success rates for asylum applications among industrialised countries.

Technical Internship Training Program [TITP] is a popular entry route for international youth to gain experience in Japanese industry, with the number of interns reaching 410,972 in 2019 (Ministry of Justice, 2020a) and the number of ex-TITP visitors overstaying their visa reaching 12,427 in January 2020 (Ministry of Justice, 2020b). The working and living conditions of technical interns are dependent on their assigned employers, as they are not allowed to move to another employer during the internship. Despite the establishment of the Organization for Technical Internship Training (OTIT) through the new Technical Intern Protection Law in 2017, there is limited protection for interns who are often faced with exploitative working conditions, inadequate salaries and verbal and physical abuses which push some to leave their internships earlier and become ‘illegal stayers’. For Bélanger et al. (2011), TITP has contributed to a structural embeddedness of irregular migration in Japan.

Nguyen, an ex-technical intern from Vietnam, is the oldest child in her family. She has two brothers with severe disabilities and elderly parents. She decided to take a technical internship in Japan because the sending organisation promised that ‘you can easily earn 150,000-200,000 JPY per month in Japan.’ She started her technical internship at a sewing company in a rural area. Her actual salary was 60,000–90,000 JPY per month after taxes, much less than what was promised. Since technical interns are bound to the company that sponsors them and her sending agency asked for a deposit to prevent her from running away, Nguyen continued her internship with the same employer for 3 years and managed only to repay her debt for coming to Japan. She was not able to make any saving. The day after her final day as an intern, the company went bankrupt. Usually, companies have to make sure their interns return to their home countries, because if someone runs away, the company might not be able to recruit new interns according to the new law enacted in November 2017. In Nguyen’s case, nobody from her former company was overseeing her return because the company had no need for technical interns anymore. Nguyen saw the opportunity and decided to stay in Japan. She went to Tokyo, aiming to make money to save and support her family in Vietnam. While in Tokyo, an acquaintance suggested her to apply for asylum:

After my visa expired, one Vietnamese female approached me and said: ‘If you apply for asylum, you can get a visa even though your visa has already expired.’ I paid 50,000 JPY to her [to help with the asylum application]. I got to know her through the internet. She said ‘Believe me’.

Eventually the application was rejected and Nguyen was detained in an immigration centre as an ‘illegal stayer’.

Tam, a Vietnamese man, had to leave his internship company in Fukuoka because of constant verbal abuse from his employer for about a year. Then he found an informal job at a factory in a town near Tokyo. His salary is lower than Japanese colleagues, but much higher than what he was paid as a technical intern. Despite having to lose his deposit for the internship, the higher salary in a new place enabled him to repay his debt and make some savings to return to Vietnam. In principle, technical interns have a right to lodge a complaint about their employers to supervision organisations who work under OTIT. However, because supervision organisations depend on payments from companies which hire technical interns, there is a shared understanding among interns that supervision organisation’s decisions are negatively biased towards them. Furthermore, the long and bureaucratic process required to lodge a complaint further deters interns from availing themselves of this instrument. There are also cases of forced return of interns following complaint procedure.

Together with technical internship, student visa is another major entry route into irregular stay. Education is a common channel for labour mobility in Japan (Liu-Farrer, 2009). Since the early 1990s, a large number of former students have overstayed their visa and become irregular migrants (Liu-Farrer, 2011; Kato, 2019). In higher education institutions and a number of professional colleges and language academies, students are allowed to work 28 h per week. In the late 1980s and throughout the 1990s, most international students derived from China and South Korea, more recently students from Vietnam have pursued this route in larger numbers (Hosogaya, 2020). In some cases students are victims of human trafficking in Japan as some brokers use the education route as a channel for the mobility of bonded unfree labour (Sasaki, 2020).

The case of Dat, a young man from Vietnamese, illustrates the education pathway into irregular status. In Dat’s words:

I couldn’t learn Japanese there. That Japanese language institution did not teach at all. They only gave us assignments. I have not even learned the basics. My Japanese is still weak. My sending organization in Vietnam had said, ‘You can study there. The Japanese language institution will look for a part-time job for you. Of course, they will educate you well.’ I wanted to learn about Japanese people and culture. I dreamed of a good future in Japan. However, I despaired after coming to Japan. I felt abandoned right after coming to Japan.

Dat’s plan was to undertake higher education in Japan after completing the Japanese language course, but his plan was jeopardised by the interference of the Japanese language school to which his visa was dependent upon. Though he took the exam to enter a hairdresser school by himself, he was ultimately unable to enroll. Frustrated for not being able to fulfil his aspiration, Dat wanted to return to Vietnam but his parents did not let him to as he first had to repay his large debt to a moneylender. As a result, he overstayed his student visa. Dat works at a food processing factory and hopes to leave as soon as he will be able to repay his debt.

In a country noteworthy for extremely low success rates for asylum applications, applying for asylum has at times been used by some as a legal yet temporary entry pathway into Japan’s labour market. Right to work for asylum claimants was granted between 2010 and 2017, over this period the country registered a growth in asylum applications which peaked in 2017 (Kato, 2019). Application for asylum has been one way for some migrants to extend their stay ‘legally’ in Japan and continue working while awaiting a decision. Asylum applications peaked in 2017, with more than a quarter of applicants being either technical interns or students. However, since 2018, Japan has stopped granting visas and work permits to most asylum seekers.^2^

### Entering in a hostile environment

The journeys that brought irregular migrants to the UK varied significantly both in terms of duration and broader significance in the biographies of interviewees. For illegal entrants, the journey is often a traumatic memory during which they had to endure extreme hardship and violence, including seeing fellow travellers dying along the route (McMahon & Sigona, 2020). The fear of not surviving the journey is central to the account of Wen Maojia, a 28 year old Chinese woman:

The lorry was transporting goods. They let me sit in a small space inside one corner, which was covered by the goods on the outside. [...] There was no light, you couldn’t see the sunshine outside … . there was some air for you to breathe, but the air was terrible inside. [...] You would have no time to think too much … There was no turning back! You’d realize that you’ve come to a point where there is no return … that you’re half way and that the only way to go is to carry on …

Differently, in the accounts of Nigerian, Jamaican, and Brazilian interviewees, especially for those who came on tourist, visitor and student visas in the first place or subsequently for family reunion, the journey is mostly narrated as a movement from A to B in which going through immigration control at the UK airport seems to be the main moment of concern for interviewees. The account of Mariana from Brazil illustrates this point:

I went to a travel agent who was recommended by a friend of mine. [...] He introduced me to this person and I was told that there was a flight in 2 weeks and I said fine. It was a shock for everybody; nobody believed I was coming in two weeks.

However, access to tourist, student and visitor visas has become increasingly difficult since the end of the 2000s with the introduction of the ‘hostile environment’ policy. The transition to more stringent control on visa is captured by Halyna, a 26 year old from Ukraine, who failed to renew her student visa because the school she was enrolled for her English language training had been blacklisted by the Home Office.

I couldn't extend my visa because of that school. It happened that there were a lot of those different schools and the Home Office was inspecting those schools. And, basically, that school where I was studying, they got into some unpleasant [things] and I was automatically stamped with refusal. And all my people I knew who studied in that school, they also were all refused [by the Home Office]. All, all, all. And I simply... I didn't have any more money for appeals so I remained here. Stayed on what I had [overstayed].

While in Japan visa overstayers and ex-technical interns make the bulk of irregular migrants, in the UK illegal entry is also a route into irregular stay. In Japan, the conditions attached the TITP and language school visa strongly limit the options available to migrants, tie then to brokers and providers and leave them financially exposed. In the UK, more stringent control on visa sponsors introduced as part of the hostile environment policy reduced the options for legal entry.

## Legal status and the infrastructures of settlement

In this section, we focus on two central aspects of migrants’ settlement experiences in Japan and the UK, namely, access to labour and work environment, and housing and living conditions and explore the extent to which lack of legal status impacts on them. We also consider migrants’ coping mechanism and ways to mitigate the impact of irregularity.

### Settling despite irregular status

High demand for low paid labour and low risk of being detected and fined for employers who recruit irregular migrants ensure that migrants can secure resources for surviving in Japan. Our interviewees mainly worked in construction, agriculture and catering business, sectors that struggle to attract Japanese labour.

Although hiring ‘illegal stayers’ became a criminal act due to revision of Immigration Control and Refugee Recognition Act in 1990, employers of irregular migrants did not fear hiring them. For one of the employers we interviewed, an ex-civil engineer who had worked with Iranian and Kurdish irregular migrants, the priority is to ensure that projects are delivered in time, not the passport of his workers.

Because of the delivery date of construction, I don’t have time to take care of the nationality of employees. I’ve had to talk with police officers a few times. We work outside and can be easily found out. Despite this, no police officer has yet arrested any of my employees.

A recent survey of foreign residents across Japan evidenced that ‘nearly a third of foreigners living in Japan say they have experienced derogatory remarks because of their background, while about 40% have suffered housing discrimination’ (Hurst, 2017). It is difficult for irregular migrants to rent a room from real estate agents. However, every informant found the place to live. Most common pattern is living with friends who have legal status and paying part of rent.

Huy, a Vietnamese ex-student lived in a dormitory surrounded by factories in Tokyo. About his landlord, he says: ‘my landlord doesn’t care about my legal status’.

ICT infrastructures such as smartphones, group chats and Facebook enable irregular migrants to build and maintain their social networks. They can easily compare their salaries and discuss employment opportunities or even find local intermediaries, who for a fee provide advice on how to prepare documents for the application for asylum in Japan. Technical interns are often connected through Facebook having undergone training together in the same sending organization in their home country. These connections, according to a Vietnamese interpreter working for a supervision organization, offer a reliable safety net for interns in case they need work or accommodation.

### Working and living as an irregular migrant in the UK

In the UK, the lack of legal status determines not only employment and housing opportunities available to migrants, but also their social networks and relationships (Sigona, 2012). The hostile environment policy has produced a more stringent and all-encompassing condition of irregularity, one where bordering practice penetrates many aspects of everyday life. The following quote from Jamie, a 31 years old man from Zimbabwe, captures the sense of vulnerability and precarity that reverberated from changes occurring to the infrastructures of settlement.

It worries me because you sort of always trying to find out what’s happening, you can have a job but you are not really secure in your job because if anything changes in terms of what they require at work, you know you are more vulnerable in that sense.

Some interviewees who lived in the UK for several years compare and contrast the current challenges they face to access housing and employment with their initial experiences. Finding employment in restaurant kitchens, for example, has become more difficult due to frequent raids by immigration police targeting ethnic restaurants and the sanctions employers may incur if irregular migrants are found working for them, including substantial fines and potentially the inclusion in a ‘name and shame’ database. Even when employment is still available, employers mitigate risks by reducing rates (Bloch et al., 2014).

Moreover, the increased emphasis on immigration status makes employees more vulnerable to exploitation by employers who often refuse to pay salaries altogether knowing that there is little room for redress for irregular migrant workers. Migrants from China reported particularly poor working conditions and instances of exploitation. However, due to their lack of status, most felt powerless and unable to speak out or take action in order to right this situation out of fear of detection and losing the job they nonetheless depended on. Xian Li explains her husband’s situation:

Sometimes he can’t get his wages at all … They just don’t give it to you … . Since you don’t have status, what can you do about it? Can you sue them?

Accommodation arrangements varied considerably among our interviewees; however, it is possible to detect some commonalities. In a few cases despite the lack of legal status, interviewees had access to some form of housing support by local councils because they, as a family with children, were deemed ‘in need’. However, the majority of the households in our sample were in privately-rented accommodation, aware that housing support comes also with increased visibility to immigration authorities. Solidarity and support from family, friends and more broadly fellow nationals are important for finding accommodation. The issue of overcrowding was mentioned in several interviews. Xian Li shares a small room with her husband and child; they cannot afford any more than this with their income.

Three of us live in one room. We placed two single beds in the room and there isn’t much space left. But what can you do about it? You have no alternative. We really can’t afford to pay more than this.

Sharing a house with members of the enlarged family is also common and often this produces tensions within the household. Subletting rooms and bedsits in rented accommodation is also common among other respondents, particularly Chinese. Typically, a property is rented by a regularly residing migrant who then sublets part of the properties to irregular migrants for a profit. This tends to be a very precarious arrangement, the duration of which depends exclusively on the contract of the primary lender, generating as a result high mobility among residents and asymmetrical power relations between regular and irregular migrants.

To summarize, regulatory migration infrastructures shape employment and housing opportunities available to migrants both in Japan and in the UK. In both countries social networks and family ties provide the infrastructure that enable irregular migrants to navigate informality and cope with precarious conditions of everyday life.

## Control and exit infrastructures: facing immigration enforcement

Similarly to other immigration countries, in Japan and the UK the immigration enforcement apparatus has expanded in response to the heighten visibility of irregular migration in public consciousness in recent years. Stricter immigration policing, including expansive use of detention and removal, has been accompanied by so-called ‘soft’ enforcement measures, with greater attention being paid to diffused controls on access to public services and means of livelihoods (see Yuval-Davis et al., 2019; Bloch & Schuster, 2005).

### Deporting migrants ‘voluntarily’

In 2018, Japan removed 9369 (Ministry of Justice, 2019), largely via voluntary or assisted return. Only 216 people were forcibly removed. While removal may not be widely used, immigration enforcement authorities can easily detain irregular migrants without any court involvement or decision. According to Global Detention Project (2020) the overall number of immigration detainees in 2019 was 22,624, with 1054 detained on a given day. Advocates have lamented the ‘policy of detaining all (*zenken shūyō shugi*)’, and length of detention. Not surprisingly, fear of detention is more widely discussed among irregular migrants than deportation.

Often, those lined up for removal are given the opportunity to negotiate and even organise return themselves. For example, Myrna, from the Philippines, and her husband, a Japanese citizen, had sought special permission for residence, however the immigration authorities did not grant her residence because Myrna had a criminal record, having entered the country on a forged passport. She also had been previously removed from Japan. However, immigration authorities proposed a deal to the couple: if Myrna returned to the Philippines voluntarily, instead of the statutory 10-year ban to enter Japan she would be offered a one-year ban. The mediation of her Japanese husband contributed to the positive outcome. Myrna was sceptical, but her husband was confident the immigration authorities would fulfil their part of the deal:

I tried to persuade Myrna to take this deal. Initially Myrna did not trust the immigration authorities. It is only one year, I told her. Why not to get this deal?

The couple even organised a farewell barbeque party for friends and relatives before her departure.

For others, like Armand, the immigration authorities were less favourable. Armand, a migrant man from the Philippines, was deported in July 2018 despite having lived in Japan for 25 years. After receiving his deportation order, to strengthen his plead to stay in Japan he had considered marrying a permanent resident Filipina. Immigration authorities tend to be tolerant to marriage cases. It looked like immigration continued provisional release every month to wait Armand’s marriage. However, the marriage didn’t occur. Then, Armand was finally detained and forcibly deported.

While forced removals are rare, detention occurs often, and causes concerns and fears among many irregular migrants. Many of the respondents mentioned being unable to sleep at night because of fear of detention, and therefore being unable to repay their debt and support their families in Japan and at home. Fernand, a migrant man from the Philippines, has a partner and two small children in Japan. He has a temporary deferral of deportation order that is renewed on a monthly basis when he goes to the immigration office. He knows that one day he may be refused renewal, and be detained and deported.

I cannot sleep at all because of fear of detention. If I am detained, how can my family survive? After extending my provisional release for a month, I can sleep for a week or so. But the next visit is coming very soon. Then I start to not be able to sleep well again.

Contingency planning is also on the card. Sukhvir, a migrant man from India, works as a carpenter on weekdays and has a part-time job at weekends to make some savings for his family to prepare for what he calls ‘the worst scenario’, that is being detained by immigration authorities and unable to support his family in Japan.

### Immigration enforcement and family life in the UK

Despite the ‘law and order’ rhetoric that underpins the hostile environment policy, recent UK estimates on the irregular migrant population (Pew Research Centre, 2019) and data on forced and assisted removal (Home Office, 2020) confirm the persistence of a sizeable irregular migrant population in the country, and that the ‘deportation gap’, that is ‘the gap between the number of people eligible for removal by the state at any time and the number of people a state actually removes (deports)’ (Gibney, 2008, p. 149), may even have expanded under hostile environment policy. According to Pew Research Centre (2019) the UK population of ‘unauthorized’ migrants is estimated between 800,000-1,200,000 at the end of 2017, while both forced and voluntary removals have been on a declining trend in the last decade.

Worries about being sent home were prevalent throughout all UK interviews, though the reasons for and strength of fear varied. This was often connected to the reasons for which people migrated in the first place and what was awaiting them in their countries of origin (Bloch et al., 2014). Another factor that influenced feelings about returning home was linked to the configuration of their family, including whether they had children born in the UK or not and where their partner was from. Fear of family separation due to different legal status and circumstances was widespread among participants. Some interviewees, worn out by the experience of ‘illegality’, were open to the possibility of return, but felt that this was not something they could impose on their children. Chez, a Jamaican mother, explains these thoughts as follows:

There have been a few times I said oh if the kids weren’t here. I would probably have gone back home already, but because they are here, and I think, they are in schools and they’re getting on...they don’t want to go back anymore.

Another factor that influenced the level of fear about being returned home was the costs invested in getting to the UK in the first place and paying off related debts, and importantly any previous experiences of arrest, detention or deportation. Sehriban, a Kurdish mother of two children, had previously been deported to Turkey together with her children. Their experiences of being picked up from their home at five in the morning by immigration officers, then detained and deported to Turkey, were traumatic and left a lasting fear of the authorities with them. Sehriban talks about the way that the UK immigration police took them from their home and then deported them:

One day the police raided the house at five in the morning and took us away to the camp. I didn’t have the psychology to cope anymore and neither did my children. I decided to go. They put us on a plane, they handcuffed my hands...A Turkish hostess came and said ‘what crime have you committed?’ It was a terrible question...

Although many mentioned that they ‘try not to think about it too much’ at the same time they would avoid as much contact with any authorities as possible. For Marcia, a Brazilian mother,

Now [after I became irregular] I am afraid of everything, in relation to needing access to health care for a serious issue, being caught by immigration at work or walking on the street, as it sometimes happens.

Liaising with the UK immigration police plays a central role in the lives of our interviewees. This can involve both being in regular contact with the authorities and avoiding contact with them altogether. In both situations, this results in serious constraints on interviewees’ mobility. Tahira, an Afghan mother of four children, has received an electronic tag and is expected to report to an immigration office on a weekly basis. Apart from not understanding why she has to comply with both these requirements, they also have a serious impact on her daily life and especially on the care of her children. On the other side of the spectrum there are those interviewees who avoid any contact with the police for fear of being picked up and deported. As Jose, a Brazilian father, explains:

I worry that you are on a bus or on the tube and suddenly someone like from the Home Office/Immigration turns up...so you always have this worry.

This has meant that Jose tries to travel as little as possible on public transport and sticks to routes that are familiar to him and where he feels safe. However, looking after children does not always allow this. They have to be taken to and picked up from school, friends’ homes, or other social activities. At the same time, Jose feels that having children in the UK means that his worry about being detected is even stronger, as now they have a life here as a family. It is especially for his children that he does not want to be detected and deported to Brazil as the children’s school is in the UK, everything they know and have is in the UK.

Fear of detection by immigration authorities also affect access to basic services, like healthcare. Few interviewees had sought or needed hospital treatment; the main exception was to give birth. Ahmad, a young man for Afghanistan, explains this in the following way:

No, I am scared to go to the hospital I always think that I will be deported. So I never go to hospital no matter how sick I am.

## Conclusion

Our approach was informed by critical scholarship on irregular migration that has highlighted the legal and social production of illegality as a historically and geographically situated process which in turn shapes the experiences and horizons of irregular migrants. However, we use migrant infrastructures to highlight the need for including a wider range of processes, spaces and actors in understanding the production and characteristics of migrants’ illegality including the situated experiences of irregular migrants. Debate on migration infrastructures has focused mostly on the role of brokerage and social networks in relation to recruitment and entry of migrant labour in destination countries. More recent theoretical advances in the field have conceptualised and typologised infrastructures, highlighting both material and immaterial components, from laws, trade arrangements and public discourses to social networks, ICTs and transport infrastructures. We have built from these insights to develop our comparative analysis of irregular migration in Japan and the UK. In doing so we have proposed a three-legged analytical framework which besides entry, considers also infrastructures of settlement and exit and how they shape the lives of irregular migrants at different stages of their migration project.

In terms of irregular migrants’ entry routes into Japan and the UK, in both countries unauthorised entry is not the main route into ‘illegality’. Overstaying temporary work, visitor visas and study visas was more common by far among our informants in both countries. In the Japan’s case, we found the technical internship, a state-supervised recruitment scheme, providing a straightforward legal entry pathway into the country however locking applicants in a position of vulnerability and dependency once in the country, one from which some eventually run away. In the UK, entry routes are more varied and less structured. Another significant difference between the two countries is that in the UK the hostile environment policy introduced in the early 2010s has tighten access to visa from many countries, increased visa cost and enhanced use of technologies to detect unauthorised entry. On the contrary, in Japan, short term visitor visa are more accessible to nationals from a wide range of countries. However, conditions imposed by sending and receiving organisations involved in the technical internship scheme and language courses may lead some migrants into ‘illegality’. Enrolment cost to sending organisations and the so-called ‘bonding deposit’ with technical internship employers create significant debt for migrants, which many find extremely difficult to pay unless they find another income, and become irregular migrants.

Precarious legal status structures the position of migrants in the labour market. In both countries the requirements on employers and landlords in terms of checking immigration status have pushed irregular migrants into the informal economy, and make them more vulnerable to exploitation.

The hostile environment policy in the UK has also had a significant impact on settlement strategies and irregular migrants’ everyday lives. It has made housing and employment conditions worse, impacting on wages and quality of accommodation. In Japan, similarly, despite the heightened demand for migrant labour, from the beginning of the 2000s the introduction of ‘The Action Plan against Crime’ and the subsequent introduction of residence cards for foreigners, together with rising populism, has meant that housing and working conditions for irregular migrants have worsened.

Finally, we examined exit infrastructures and how they affect migrant lives and expectations. Enforcement practices are different, law and order rhetoric is more visible in the UK case, while in both countries deportation figures show nonetheless a significant ‘deportation gap’. Migrants’ detention is becoming more common in Japan, but removal is mostly voluntary and there is room for migrants to negotiate some of the terms of their removal.

In conclusion, if we are to understand the contemporary phenomenon of irregular migration in Japan and the UK and how it shapes the everyday lives of irregular migrants, it is not enough to focus on the actors and mechanisms that facilitate and mediate entry. We argue that, given the persistence and in some cases widening of the ‘deportation gap’, attention should be paid to the infrastructures of settlement and exit. The former defines not only the spaces and opportunities for irregular migrants’ settlement but also enable us to locate migrant agency and define the contour of their contingent and precarious political subjectivities. The latter illuminates a core feature in the life of irregular migrants, namely the fear of detection and deportation, helping us to explain specific configurations and manifestations of deportability.

## Data Availability

The datasets that support the findings of this study are not publicly available due to them containing information that could compromise research participant consent. Data on the UK was previously published in No Way Out, No Way In (Sigona & Hughes, 2012) and No Right to Dream (Bloch et al., 2009).

## Footnotes

1 Suzuki. W. Abe’s cabinet approves plan to accept more foreign workers (2018). 2 November. https://asia.nikkei.com/Spotlight/Japan-immigration/Abe-s-cabinet-approves-plan-to-accept-more-foreign-workers2

2 The chance of receiving refugee status in Japan is very low; for example, in 2019 only 44 people were granted refugee status out of 10,375 applicants (Ministry of Justice, 2020c).
